# Inner Ear Hair Cell Protection in Mammals against the Noise-Induced Cochlear Damage

**DOI:** 10.1155/2018/3170801

**Published:** 2018-07-15

**Authors:** Muhammad Waqas, Song Gao, Iram-us-Salam, Muhammad Kazim Ali, Yongming Ma, Wenyan Li

**Affiliations:** ^1^Department of Biotechnology, Federal Urdu University of Arts, Science and Technology, Gulshan-e-Iqbal Campus, Karachi, Pakistan; ^2^Key Laboratory for Developmental Genes and Human Disease, Ministry of Education, Institute of Life Sciences, Southeast University, Nanjing 210096, China; ^3^Department of Otolaryngology, Affiliated People's Hospital of Jiangsu University, Zhenjiang 212002, China; ^4^Karachi Institute of Biotechnology and Genetic Engineering (KIBGE), University of Karachi, Karachi 72570, Pakistan; ^5^ENT Institute and Department of Otorhinolaryngology of the Affiliated Eye and ENT Hospital, State Key Laboratory of Medical Neurobiology, Fudan University, Shanghai 200031, China

## Abstract

Inner ear hair cells are mechanosensory receptors that perceive mechanical sound and help to decode the sound in order to understand spoken language. Exposure to intense noise may result in the damage to the inner ear hair cells, causing noise-induced hearing loss (NIHL). Particularly, the outer hair cells are the first and the most affected cells in NIHL. After acoustic trauma, hair cells lose their structural integrity and initiate a self-deterioration process due to the oxidative stress. The activation of different cellular death pathways leads to complete hair cell death. This review specifically presents the current understanding of the mechanism exists behind the loss of inner ear hair cell in the auditory portion after noise-induced trauma. The article also explains the recent hair cell protection strategies to prevent the damage and restore hearing function in mammals.

## 1. Introduction

The inner ear is the most incredible and sophisticated organ of the body. It connects the personnel with the outer world in the form of hearing. The hearing loss is referred to as the most common sensory disorder that affects all age groups of the world population. The complex architecture of the mammalian organ of Corti makes it more susceptible to damage and is difficult to revert back into its native form [1, 2]. Although the neonatal cochlea holds the potential to form new hair cells by transforming the supporting cells (such as Lgr5+ cells) into the hair cells in the apical till basal region [3–6]. This capability completely sheds off in the adult sensory epithelium. Lack of understanding of the mechanical sound voice has a massive impact on a person's ability to communicate and deal with the normal and emergency life situations. It badly affects the patient's mental and physical health as well as makes their life isolated and depressed [7–10]. Such people are more in danger of accidental injuries than others and are completely relying on their attendant [11].

Sensorineural hearing loss (SNHL) is referred to as the most common type of hearing disorder occurs due to the damage or loss of the hair cells, the neuron-hair cell synapses, and/or degeneration of neurons. The SNHL is not completely recoverable due to the lack of self-regenerative capacity of HCs and SGNs. The patients having SNHL may be provided with the hearing aids, and in case of severe to profound hearing loss, the patients have the only option of cochlear implants [12]. However, besides the advancements in the engineering, surgical, and pharmaceutical operations, normal hearing function yet not completely be restored using hearing devices.

There are multiple etiologies of SNHL. At any age, the foremost reasons for hearing loss are genetic and the environmental factors. The main causes of SNHL are degenerative processes associated with aging, gene mutations, noise exposure, and the use of therapeutic drugs that have ototoxic side effects [13–16]. Interestingly, the noise and the ototoxicity are actually the consequences of men made technological advancements and do not really exist in nature. Other etiologies include the autoimmune disorder, head injury, and the hair cell overstimulation [17–21]. Exposure to intense noise results in the irreversible damage to hair cells via different cellular mechanisms. In this review, we aim to discuss the different mechanisms of hair cell damage and highlight the recent findings as well as possible strategies for hair cell protection against the noise-induced hearing loss.

## 2. Mechanism of Hair Cell Loss in Mammals after Noise-Induced Trauma

Stereociliary bundles found on the surface of hair cells are more susceptible to mechanical damage. The exposure to intense noise causes direct mechanical disruption of stereociliary structure and disrupts the normal cellular organization of the organ of Corti [22–24]. However, the deepest level of damage is not only because of intense mechanical sound but also depends on different cellular pathways involved in hair cell growth.

### 2.1. Noise-Induced Oxidative Stress

**Figure 1:**
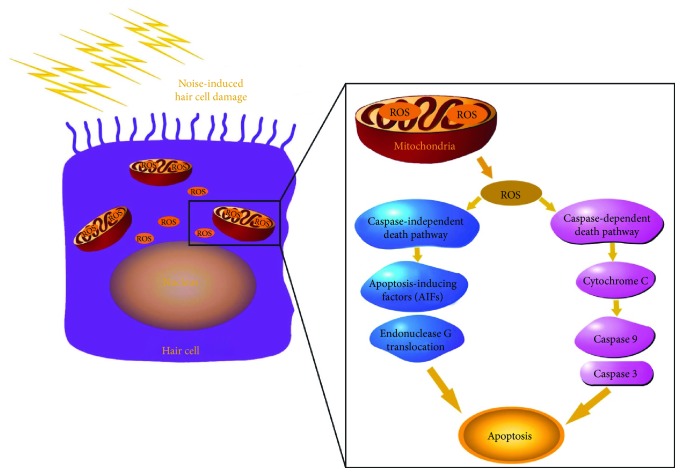
Schematic of the generation of reactive oxygen species (ROS) along with the activation of caspase-mediated and independent death pathways in hair cell after noise-induced oxidative stress.

The reactive oxygen species (ROS) are observed in the hair cells after the acoustic overexposure and exist there for about 10 days [25]. The ROS are produced in the cell mitochondria, and disturbance in the integrity of mitochondria may result in the production and continuous release of ROS in the cell cytoplasm [26, 27]. The generation of reactive oxygen species and the increased metabolic activity in the hair cells after noise-induced ototoxicity have been reported to create hair cell loss (Figure 1) [28–31]. The reactive nitrogen species (RNS) also accumulate in the hair cells after being exposed to loud voices [32, 33]. Both the ROS and RNS have stimulated caspase-mediated apoptotic cell death pathways in the cochlea [30, 34]. Besides, ROS formation also promotes inflammation and generates proinflammatory cytokines such as interleukin (IL) 6 and [35, 36], tumor necrosis factor-*α* (TNF-*α*) [37, 38].

### 2.2. Caspase- and JNK-Dependent Hair Cell Death Pathways

Two complex signaling pathways are commonly involved in noise-induced hair cell loss that are known as intrinsic and extrinsic cell death signaling pathways. In the cochlea exposed to intense noise, the extracellular stimuli initiate the extrinsic cell death signaling pathway by inducing the transmembrane death receptors. These receptors activate the caspase 8, which further triggers the distinct downstream signaling pathway leading to the activation of caspase 3 that mediates apoptosis in the outer hair cells [39]. The intrinsic death pathway starts in the outer hair cells due to the modifications in the permeability of mitochondrial membrane that stimulates the caspase 9 and releases cytochrome c from mitochondria, thus induces programmed cell death [39, 40]. Together with caspases, the receptor-interacting protein kinase (RIP) is also implicated in the activation of necrotic cell death pathways in the outer hair cells of adult mice exposed to loud noise [41]. Another study has shown that after noise trauma, the c-Jun *N*-terminal kinase (JNK)/mitogen-activated protein kinase (MAPK) also induces a mitochondrial cell death pathway through the stimulation and translocation of Bax and procaspases, release of cytochrome c from mitochondria into the damage cell cytoplasm, and lastly, the cleavage of fodrin by activating caspases [42]. A recent work of Fuentes-Santamaría et al. also determined the permanent hearing threshold shift in response to loud noise overexposure. This shift simultaneously occurred with the outer hair cell loss, upregulation of prestin, and microglial activation. The authors also observed that the TNF-*α* and interleukin 1*β* were upregulated by the microglia, fibrocytes, and neuronal cells at different time points in the noise-exposed cochlea [43] suggesting that there is an involvement of complex interplay among the different cytokine-producing cells that might be responsible for cochlear pathophysiology in the noise-exposed cochlea.

### 2.3. Caspase-Independent Cell Death Pathway

Caspase-independent apoptotic pathway is also involved in the hair cell loss. After the exposure to loud noise, the mitochondria participate in the apoptosis by releasing the apoptosis-inducing factors (AIFs) and endonuclease G (EndoG) through the outer mitochondrial membrane into the hair cell cytoplasm. EndoG translocates to cell nucleus in order to initiate apoptosis while the AIFs may not directly be involved in apoptosis but act as a redox factor in return to noise-induced oxidative stress [44]. However, Han et al. reported that both AIF and EndoG were translocated to the nuclei and participated in the hair cell death [45]. The tumor necrosis factor-*α* (TNF-*α*) pathway has also been activated in the noise-induced damage model [34]; however, it is still unclear whether the pathway activation is specifically in the hair cells. According to the study of Bohne et al., three different death pathways were observed in the outer hair cells on the basis of their morphological characteristics after noise-induced auditory damage. Among them, two were the oncotic (swollen cell following rupture) and apoptotic (programmed cell death) pathways whereas in the third death pathway, the outer hair cells lose their basolateral cell membrane but maintain their cytoplasm with cellular debris intact in a cylindrical frame structure [46].

### 2.4. Excessive Calcium Accumulation in Hair Cells

The acoustic overexposure results in an increase accumulation of free calcium ions in the outer hair cells that enter through the L-type calcium channels and cell intracellular stores such as the mitochondria and endoplasmic reticulum [47, 48]. These free calcium ions independently activate both the necrotic and apoptotic pathways in outer hair cells without any ROS formation [48, 49]. However, the accumulation of calcium after acoustic overexposure in the outer hair cells stimulates the mitochondria-mediated cell death pathways through activation of Bcl-2-associated death promoters (BAD) by calcium-dependent phosphatase calcineurin [50]. This study suggests that the translocation of BAD to the mitochondria of diminishing outer hair cells is an indicator of the activation of its proapoptotic activity.

These studies highlight the possible mechanisms of hair cell loss in a noise-induced cochlear damage model. Besides the fact that the oxidative stress and the activated death pathways after acoustic overexposure detrimentally affect the cochlea, it takes a longer duration to degenerate hair cells after acoustic injury, suggesting that there is a possibility to interrupt this diminishing process in the mammalian cochlea.

### 2.5. The Genetics of NIHL and the Noise-Induced Synaptopathy

Genetic factors may contribute to the development of noise-induced hearing loss. The individual humans and animals displayed a variation in the susceptibility to noise-induced damage even under controlled conditions. This difference in susceptibility may be influenced by the genetic factors. For the last two decades, several genetic studies were performed to identify the NIHL susceptibility genes and among them various NIHL susceptibility genes have been known to involved in different cellular pathways such as the genes involved in the potassium recycling pathway (Kcnq1, Kcnq4, kcne1, Kcnj10, Gjb1, Gjb2, and Gjb4) [51, 52], oxidative stress gene (Sod2, Cat, Gstm1, and Pon2) [53, 54], heat shock protein genes (Hsp70) [55], and monogenic deafness genes (Myh14 and Pcdh15) [56]. The variation in these genes has shown to be associated with the susceptibility to noise-induced hearing loss in different populations [57]. Similarly, several studies in the transgenic mice model showed that the deficit in different cellular pathway genes increases the susceptibility of the inner ear to acoustic overexposure. Many homozygous and heterozygous mice, including Cdh23 [58], Pmca2 [59], Sod1 [60], Gpx1 [61], Trpv4 [62], and Hsf1 [63, 64] knockout mice, have been shown to be more sensitive to noise-induced hearing loss than the other wild-type strains.

Apart from this, recent studies also highlight that the synapse degeneration in the inner ear is another key contributor of NIHL. The synapsis between the inner hair cells and spiral ganglion neurons is more prone to cellular damage [65]. Glutamate excitotoxicity and calcium signaling pathways are considered as a candidate in cochlear synaptopathy. The excessive release of glutamate results in a synaptic destruction between inner hair cells and spiral ganglion neurons [66]. This excessive glutamate concentration further leads to the huge influx of calcium, sodium, and potassium ions into the spiral ganglion neurons that ultimately swell and damage the synaptic structures [67–69]. Moreover, L-type and T-type calcium channels also participated to excessive calcium influx after noise-induced damage [70, 71]. However, the role of different signaling pathways and the exact mechanism of cochlear synaptopathy are not completely understood yet and need further investigations in the future.

## 3. Strategies for Hair Cell Protection in Mammals against the Noise-Induced Hearing Loss

Various strategies have been defined to protect inner ear hair cells from acoustic damage. Some of the useful external protective measures are to reduce the exposure of loud noise by eliminating the basic source of the noise. However, it is not possible to take this measure in all the conditions, particularly in the areas of high noise pollution such as in the industrial world. Therefore, the use of hearing protection devices is recommended and in somewhat effective to reduce the acoustic damage. Multiple studies have suggested the use of hearing protection devices that significantly reduce the loud noise exposure, thus minimizing the risk of hearing loss in the industrial workers [72–74]. However, the use of hearing protection devices sometimes creates a barrier in the communication and discomfort for the user if the device is not completely fit externally.

Besides the external measures of hearing protection, the otoprotective treatment on a cellular level is mainly focused for the prevention of hair cell loss and induction of the self-repair mechanism to restore auditory function. The most effective strategies used to prevent hair cell loss after noise-induced damage are: (1) use of antioxidants, (2) inhibition of programmed cell death pathways, (3) anti-inflammatory therapies, and (4) neurotrophic factors.

### 3.1. Antioxidant Treatment

Antioxidants are the potential therapeutics used to protect inner ear hair cells from acoustic damage. The oral administration of antioxidant drugs such as 4-hydroxy-alpha-phenyl-*tert*-butylnitrone (4-OHPBN) and N-acetyl-L-cysteine (NAC) after acoustic overexposure significantly reduces the noise-induced hearing loss [75]. Another study reported the use of two antioxidants (disodium 2,4-disulfophenyl-N-*tert*-butylnitrone and NAC) in combination that also protected the hair cells and afferent neurites from noise-induced damage and preserved the cochlear structural components [76]. In addition, the prior studies also reported that the NAC is an effective antioxidant that provides otoprotection against the noise-induced hearing loss in animal models [77–81]. Similarly, multiple studies in humans also reported the little significant and protective effects of NAC on hearing preservation [82–84]. The oral administration of NAC during the continuous acoustic overexposure prevents the noise-induced temporary threshold shift (TTS) following 14 days of treatment as compared to the control group [82–84]. However, Kramer et al. demonstrated that before the loud noise exposure, the oral administration of NAC alone does not have any significant otoprotective effects on the inner ear [83]. Kopke et al. worked on a large military group exposed to loud noise for 16 days and observed 6-7% reduction in the hearing threshold shift after daily oral administration of NAC [85]. Collectively, these studies highlight the beneficiary effects of NAC against the noise-induced hearing loss. Moreover, some other antioxidants are also effective to prevent the noise-induced trauma, such as synthetic organoselenium drug ebselen [86], coenzyme Q10 [87], resveratrol [88], glutathione [89], ginseng [82], D-methionine [90], and vitamins A, C, E, and B12 [91–95]. The detailed clinical trials of different pharmaceutical agents including antioxidant, against the noise-induced hearing loss, are thoroughly reviewed recently [96, 97]. These antioxidants are still in their preliminary trial phases and must subjected to further investigation in the future.

### 3.2. Inhibition of Programmed Cell Death Pathways

Manipulation of intrinsic cell death cascades using different antiapoptotic inhibitors is also a promising strategy to protect hair cells after the noise-induced hearing loss. Multiple studies have shown the activation of MAPK/JNK pathways in cellular stress response. The blocking of this pathway using the JNK inhibitory molecules in the animal model provides significant protection against the acoustic trauma [98, 99]. Similarly, the administration of the JNK inhibitor through the round window prevents hair cell death caused by the acoustic overexposure and restores hearing in an animal model in a dose dependent manner [42]. The sound trauma could potentially be minimized by the otoprotective peptide AM-111 that is also a JNK inhibitor [100, 101]. The systemic or local administration of AM-111 after impulse noise exposure provides significant protection against the noise-induced hearing loss [102]. Likewise, the subcutaneous administration of CEP-1347 (a derivative of indolocarbazole K252a and a JNK pathway inhibitor) has shown the less hearing threshold shift in the guinea pig exposed to noise [103]. The post sound exposure treatment of retinoic acid (a potent JNK pathway inhibitor) for five days in mice showed a reduced hearing threshold shift and hearing deterioration [91]. Together, these studies suggested that the use of apoptotic inhibitor is a potential therapeutic intervention in noise-induced hearing loss; however, further clinical trials are needed to form a combinative antiapoptotic strategy to treat noise-induced hearing loss.

### 3.3. Anti-Inflammatory Agents

Several types of anti-inflammatory drugs have been reported to rescue the hearing deterioration in the inner ear induced by the sound overexposure. Particularly, the use of steroids such as dexamethasone and dehydroepiandrosterone reduces the noise-induced trauma in the guinea pig and mice models [104–107]. The higher intratympanic dose administration of dexamethasone efficiently preserves the hearing in mice than the intraperitoneal administration against the noise trauma. The intratympanic administration is more effective for the efferent terminal outer hair cell synapses, while intraperitoneal administration protects the organ of Corti in a mouse model suggesting that the otoprotective effects are different if the route and dose of administration are changed [108]. As compared to intratympanic administration of steroids alone, the early concurrent administration of intratympanic steroid injections and systemic steroids preserves the hearing capability of patients more appropriately after sound trauma caused by the gunshot noise [109]. Overall, these studies highlight the effective concurrent intratympanic and systemic steroid treatment against the acoustic damage that protects the hearing and structural integrity of the cochlea. However, the long-term use of steroids may cause several adverse effects on human body.

### 3.4. Neurotrophin-3 (NT-3) and Brain-Derived Neurotrophic Factor (BDNF)

Although the neurotrophins are the key regulators for differentiation, survival, and maintenance of neuronal cells, several studies have reported their otoprotective role against the noise-induced hearing loss [110–113]. Neurotrophin-3 (NT3) and brain-derived neurotrophic factor (BDNF) are well known to participate in the development and establishment of hair cell ribbon synapses in the inner ear [111]. After noise-induced damage, NT3 expression by associated supporting cells promotes the ribbon synapse regeneration and restores their function in the cochlea [110, 111]. Surprisingly, a single dose of neurotrophins (NT3 + BDNF) delivers through the round window protects the hair cell and lessens the synaptopathy after the noise-induced trauma in guinea pigs [112]. In summary, these studies explain the potential therapeutic use of neurotrophins in the animal model. However, further research is still required to explore the potential and study the long-term effects of neurotrophins in the human model.

## 4. Future Perspective

It is very fascinating to observe that several strategies and drugs have been discovered to protect the inner ear hair cells from acoustic damage. A common strategy in recent years appears to target and manipulate the programmed cell death pathways and involves the use of antioxidants to control the oxidative stress in hair cells. After noise-induced ototoxic damage, there are different signaling pathways activated in the cochlea that induce hair cell death. The interruption in one of these death signaling cascades by specific inhibitor might not be that effective to rescue the auditory function until the multiple drugs or molecular inhibitors are not used in combination. The cellular cascades are interlinked with each other, and there is a possibility that the inhibition of one pathway with specific inhibitor might result in the activation of other cell death pathways in hair cells. Thus, a synergistic approach would be more beneficial to restore the hearing loss.

As reviewed above, to study the noise-induced hearing loss in the animal model, the researchers used various approaches such as different animal species, sound intensity, frequency spectrum, and continuous or impulse noise. This sometime creates a conflict among the results of some drug studies that have found to be otoprotective by one group while reported as ineffective by the other group. The most probable reason for these conflicts is the difference between genetic backgrounds of different animal species that have different sensitivities to intense sound; thus, there is a possibility for different responses of same drug in different animal models. It is important to understand that the majority of animal studies showed a statistically significant impact when the protective drug reduces the hearing loss by 2-3 decibels (dB). However, in humans, this shift should be more than 10 dB to be effective for the auditory performance, and the hearing below this level may have a very less impact on person hearing capability. Therefore, in the future, it is important to first perform the otoprotective therapeutic experiments in different animal models with different acoustic exposure conditions before taking it into the clinical trials.

## 5. Conclusion

In recent years, research on the noise-induced hearing loss is focused in order to develop various therapeutic strategies for appropriate protection of hair cells from any damage and to restore the auditory function after acoustic trauma. The momentum builts up by these studies on the effectiveness of different otoprotective agents such as the antioxidants, anti-inflammatory agents, and neurotrophic factors, and manipulation of the intrinsic cell death pathways in hair cells will likely drive the complete development of therapeutic interventions for restoring the noise-induced hearing loss in the future. However, identification of the optimal conditions such as the dose regimen, effective route of administration, and timings for new drugs and their synergistic plans to treat sound-induced hearing loss in patients is important to be focused in the future experiments.
